# Supplementary material for: Origin of long-lived quantum coherence and excitation dynamics in pigment-protein complexes
Source: Sci Rep. 2016 Nov 23;6:37629. doi: 10.1038/srep37629 (PMC5120302; doi:10.1038/srep37629)
Supplement: Supplementary Information [file srep37629-s1.pdf]

Origin of long-lived quantum coherence and excitation dynamics in pigment-protein complexes: Supplementary Information

Zhedong Zhang[1,3] **and Jin Wang**[1,2,4,*]

[1]Department of Physics and Astronomy, SUNY Stony Brook, Stony Brook, NY 11794, USA
[2]Department of Chemistry, SUNY Stony Brook, Stony Brook, NY 11794, USA
[3]Department of Chemistry, University of California Irvine, Irvine, CA 92697, USA
[4]State Key Laboratory of Electroanalytical Chemistry, Changchun Institute for Applied Chemistry, Chinese Academy of Sciences, Changchun, Jilin 130022, P. R. China
[*]jin.wang.1@stonybrook.edu

ABSTRACT

In this supplementary information, we will provide more details on the effective theory and coherence dynamics. Further results on the coherence dynamics of the models without considering the incoherent environment will be also included.

Renormalization of system-bath interaction by polaron

With the form of generating function S

$$S = \sum_{m=1}^{N} \sum_{\mathbf{q},s} f_{\mathbf{q}s} \gamma_m a_m^\dagger a_m \left(b_{-\mathbf{q},s}^\dagger - b_{\mathbf{q}s}\right) \tag{S0}$$

we can obtain the following commutators

$$\begin{aligned}
[S,H] &= \sum_{i<j} \sum_{\mathbf{q},s} f_{\mathbf{q}s} J_{ij} (\gamma_i - \gamma_j) (a_i^\dagger a_j - a_j^\dagger a_i) \left(b_{-\mathbf{q},s}^\dagger - b_{\mathbf{q}s}\right) - \sum_{m=1}^{N} \sum_{\mathbf{q},s} f_{\mathbf{q}s} \hbar\omega_{\mathbf{q}s} \gamma_m a_m^\dagger a_m \left(b_{-\mathbf{q},s}^\dagger + b_{\mathbf{q}s}\right) \\
&\quad - 2 \sum_{m,n=1}^{N} \sum_{\mathbf{q},s} f_{\mathbf{q}s}^2 \hbar\omega_{\mathbf{q}s} \gamma_m \gamma_n a_m^\dagger a_m a_n^\dagger a_n \\
[S,[S,H]] &= \sum_{i<j} J_{ij} (\gamma_i - \gamma_j)^2 \left(a_i^\dagger a_j + a_j^\dagger a_i\right) \left(\sum_{\mathbf{q},s} (b_{-\mathbf{q},s}^\dagger - b_{\mathbf{q}s})\right)^2 + 2 \sum_{m,n=1}^{N} \sum_{\mathbf{q},s} f_{\mathbf{q}s}^2 \hbar\omega_{\mathbf{q}s} \gamma_m \gamma_n a_m^\dagger a_m a_n^\dagger a_n \\
[S,[S,[S,H]]] &= \sum_{i<j} J_{ij} (\gamma_i - \gamma_j)^3 \left(a_i^\dagger a_j - a_j^\dagger a_i\right) \left(\sum_{\mathbf{q},s} (b_{-\mathbf{q},s}^\dagger - b_{\mathbf{q}s})\right)^3 \\
&\quad \vdots \qquad\qquad\qquad \vdots \\
[S,[S,\cdots[S,H]]\cdots] &= \sum_{i<j} J_{ij} (\gamma_i - \gamma_j)^M \left(a_i^\dagger a_j + (-1)^M a_j^\dagger a_i\right) \left(\sum_{\mathbf{q},s} (b_{-\mathbf{q},s}^\dagger - b_{\mathbf{q}s})\right)^M
\end{aligned} \tag{S1}$$

Hence from the Baker-Campbell-Hausdorff formula one has the new Hamiltonian

$$\begin{aligned}\tilde{H} &= e^{S}He^{-S} = H + [S,H] + \frac{1}{2!}[S,[S,H]] + \frac{1}{3!}[S,[S,[S,H]]] + \cdots \\ &= \sum_{i=1}^{N}\Big(\varepsilon_i - \gamma_i^2\sum_{\mathbf{q},s} f_{\mathbf{q}s}^2\hbar\omega_{\mathbf{q}s}\Big)a_i^\dagger a_i + 2\sum_{i<j}\sum_{\mathbf{q},s} f_{\mathbf{q}s}^2\hbar\omega_{\mathbf{q}s}\gamma_i\gamma_j a_i^\dagger a_j^\dagger a_i a_j + \sum_{\mathbf{q},s}\hbar\omega_{\mathbf{q}s}b_{\mathbf{q}s}^\dagger b_{\mathbf{q}s} \\ &\quad + \sum_{i<j} J_{ij}\left[\prod_{\mathbf{q},s} e^{(\gamma_i-\gamma_j)f_{\mathbf{q}s}(b^\dagger_{-\mathbf{q},s}-b_{\mathbf{q}s})}a_i^\dagger a_j + \prod_{\mathbf{q},s} e^{-(\gamma_i-\gamma_j)f_{\mathbf{q}s}(b^\dagger_{-\mathbf{q},s}-b_{\mathbf{q}s})}a_j^\dagger a_i\right]\end{aligned} \tag{S2}$$

As the quasi-resonance between excitons and discrete vibrational modes gives rise to the strong coupling, these modes should be separated from other phonon modes which are off-resonance to the excitons. These modes being off-resonance to excitons can then be reasonably treated as environment. Mathematically this is realized by separating the product of phonons in Eq.(S2) and we further do the expansions up to the 1st order of coupling strength between the system and the remaining phonon modes

$$\begin{aligned}&\prod_{\mathbf{q},s} e^{(\gamma_i-\gamma_j)f_{\mathbf{q}s}(b^\dagger_{-\mathbf{q},s}-b_{\mathbf{q}s})} = \prod_{n} e^{(\gamma_i-\gamma_j)\lambda_n(d_n^\dagger-d_n)}\prod_{\mathbf{q},s}{}' e^{(\gamma_i-\gamma_j)f_{\mathbf{q}s}(b^\dagger_{-\mathbf{q},s}-b_{\mathbf{q}s})} \\ &e^{\pm(\gamma_i-\gamma_j)f_{\mathbf{q}s}(b^\dagger_{-\mathbf{q},s}-b_{\mathbf{q}s})} \simeq 1 \pm (\gamma_i-\gamma_j)f_{\mathbf{q}s}(b^\dagger_{-\mathbf{q},s}-b_{\mathbf{q}s})\end{aligned} \tag{S3}$$

which gives

$$\prod_{\mathbf{q},s} e^{(\gamma_i-\gamma_j)f_{\mathbf{q}s}(b^\dagger_{-\mathbf{q},s}-b_{\mathbf{q}s})} \simeq \prod_{n} e^{(\gamma_i-\gamma_j)\lambda_n(d_n^\dagger-d_n)}\left[1 + (\gamma_i-\gamma_j)\sum_{\mathbf{q},s} f_{\mathbf{q}s}(b^\dagger_{-\mathbf{q},s}-b_{\mathbf{q}s})\right] \tag{S3.5}$$

Substituting Eq.S3.5 into Eq.(S2) and using the identity

$$e^{A+B} = e^{A}e^{B}e^{-[A,B]/2} \text{ when } [A,[A,B]] = [B,[A,B]] = 0 \tag{S3.6}$$

gives rise to the effective Hamiltonian in Eq.(2) in main text.

Coherence Dynamics

The total Hilbert space of system in our model is $\mathscr{H} = \mathscr{H}_{ele}^{(1)} \otimes \mathscr{H}_{ele}^{(2)} \otimes \mathscr{H}_{vib}$, where the basis is ordered as

$$|1\rangle = |0,m\rangle,\ |2\rangle = |0,m+1\rangle,\ |3\rangle = |A,m\rangle,\ |4\rangle = |A,m+1\rangle,\ |5\rangle = |B,m\rangle,\ |6\rangle = |B,m+1\rangle \tag{S4}$$

which leads to the matrix form of Hamiltonian for exciton+vibron

$$H_S = \begin{pmatrix} m\hbar\omega & 0 & 0 & 0 & 0 & 0 \\ 0 & (m+1)\hbar\omega & 0 & 0 & 0 & 0 \\ 0 & 0 & \varepsilon_A + m\hbar\omega & \lambda\hbar\omega\sqrt{\frac{m+1}{2}} & J & 0 \\ 0 & 0 & \lambda\hbar\omega\sqrt{\frac{m+1}{2}} & \varepsilon_A + (m+1)\hbar\omega & 0 & J \\ 0 & 0 & J & 0 & \varepsilon_B + m\hbar\omega & -\lambda\hbar\omega\sqrt{\frac{m+1}{2}} \\ 0 & 0 & 0 & J & -\lambda\hbar\omega\sqrt{\frac{m+1}{2}} & \varepsilon_B + (m+1)\hbar\omega \end{pmatrix} \tag{S5}$$

thus the orthogonal matrix U is of block form according to the form of H_S above. In the new basis where H_S is diagonalized, the excitonic coherence is of the form

$$\begin{aligned}\langle \underline{5}|\rho|\underline{3}\rangle &= \sum_{i=3}^{D}\sum_{j=3}^{D} U_{i5}U_{3j}^T\langle i|\rho|j\rangle = \sum_{n=3}^{D} U_{3n}^T U_{n5}\rho_{nn} \\ &\quad + \left(U_{35}U_{35}^T\rho_{35} + U_{55}U_{33}^T\rho_{53} + U_{45}U_{36}^T\rho_{46} + U_{65}U_{34}^T\rho_{64}\right),\ \textit{electronic} \\ &\quad + \left(U_{35}U_{34}^T\rho_{34} + U_{45}U_{33}^T\rho_{43} + U_{55}U_{36}^T\rho_{56} + U_{65}U_{35}^T\rho_{65}\right),\ \textit{vibrational} \\ &\quad + \left(U_{35}U_{36}^T\rho_{36} + U_{65}U_{33}^T\rho_{63} + U_{45}U_{35}^T\rho_{45} + U_{55}U_{34}^T\rho_{54}\right),\ \textit{mixed}\end{aligned} \tag{1}$$

and others components can be expressed by the similar manner, i.e., $\langle\underline{5}|\rho|\underline{4}\rangle$, $\langle\underline{2}|\rho|\underline{1}\rangle$... In such basis, the dipole operator of the system is

$$\sum_{n=1}^{2}\sum_{m=2,4}\sigma^-_{n,n+m}+\sigma^+_{n,n+m}=\sum_{i=1}^{2}\sum_{j=3}^{D}\left(U^T_{j,i+2}+U^T_{j,i+4}\right)|i\rangle\langle\underline{j}|+\text{h.c.}=\sum_{i=1}^{2}\sum_{j=3}^{D}\sum_{\bar{\omega}_{ij}=\pm\omega_{ij}}A_{ij}(\bar{\omega}_{ij}) \tag{S7}$$

where $|\underline{\mu}\rangle=\sum_{\lambda=3}^{D}U^T_{\mu\lambda}|\lambda\rangle$ and the underline denotes the basis diagonalizing H_S. $\hbar\omega_{ij}=E_j-E_i$

$$\begin{aligned}&A_{ij}(\bar{\omega}_{ij}>0)=(U^T_{j,i+2}+U^T_{j,i+4})|i\rangle\langle\underline{j}|,\ A^\dagger_{ij}(\bar{\omega}_{ij}>0)=(U_{i+2,j}+U_{i+4,j})|\underline{j}\rangle\langle i|\\&A_{ij}(\bar{\omega}_{ij}<0)=(U_{i+2,j}+U_{i+4,j})|\underline{j}\rangle\langle 1|,\ A^\dagger_{ij}(\bar{\omega}_{ij}<0)=(U^T_{j,i+2}+U^T_{j,i+4})|i\rangle\langle\underline{j}|\end{aligned} \tag{S8}$$

which leads to the following form of the interaction between system and radiations in the interaction picture

$$\begin{aligned}&\tilde{H}^{(1)}_{int}(t)=\sum_{i=1}^{2}\sum_{j=3}^{D}\sum_{\bar{\omega}_{ij}=\pm\omega_{ij}}A_{ij}(\bar{\omega}_{ij})e^{-i\bar{\omega}_{ij}t}\otimes B(t)\\&B(t)=\sum_{\mathbf{k},p}g_{\mathbf{k}p}\left(a_{\mathbf{k}p}e^{-i\omega_{\mathbf{k}p}t}+a_{-\mathbf{k},p}e^{i\omega_{\mathbf{k}p}t}\right)\end{aligned} \tag{S9}$$

and the correlation function of radiation environment

$$\langle B(t)B(t-s)\rangle=\sum_{\mathbf{k},p}g^2_{\mathbf{k}p}\left[(n^{T_1}_{\mathbf{k}p}+1)e^{-i\omega_{\mathbf{k}p}s}+n^{T_1}_{\mathbf{k}p}e^{i\omega_{\mathbf{k}p}s}\right] \tag{S10}$$

Physically the strong interactions between excitons and discrete vibrational modes owing to the frequency-match, leads to the comparable time scales between the vibrational modes and excitons, which subsequently require us to include the dynamics of these vibrational modes together with the excitons. Thereby the remaining modes consisting of low-energy fluctuations can be reasonably treated as environment, which is in weak coupling to the excitons due to the frequency-mismatch. Hence based on perturbation theory, the whole solution of density operator can be written as $\rho_{SR}=\rho_S(t)\otimes\rho_R(0)+\rho_c(t)$ with the traceless term in higher order of system-bath interaction. Because the time scale associated with the environmental correlations is much smaller than the time scale of the system over which the state varies appreciably, the QME for the reduced density matrix of the systems is of the Redfield form

$$\begin{aligned}\frac{d\rho}{dt}&=\frac{i}{\hbar}[\rho,H_S]+\mathscr{D}(\rho)\\&=\frac{i}{\hbar}[\rho,H_S]+\frac{1}{\hbar^2}\text{Tr}_Be^{-iH_0t/\hbar}\int_0^t\text{d}s\left[\tilde{H}_{int}(t-s)\tilde{\rho}(t)\rho_R(0)\tilde{H}_{int}(t)-\tilde{H}_{int}(t)\tilde{H}_{int}(t-s)\tilde{\rho}(t)\rho_R(0)\right]e^{iH_0t/\hbar}+\text{h.c.}\end{aligned} \tag{S11}$$

and the dissipation contributed by the radiations reads

$$\begin{aligned}\mathscr{D}^{T_1}(\rho)&=\frac{1}{\hbar^2}\text{Tr}_Be^{-iH_0t/\hbar}\int_0^t\text{d}s\left[\tilde{H}^{(1)}_{int}(t-s)\tilde{\rho}(t)\rho_R(0)\tilde{H}^{(1)}_{int}(t)-\tilde{H}^{(1)}_{int}(t)\tilde{H}^{(1)}_{int}(t-s)\tilde{\rho}(t)\rho_R(0)\right]e^{iH_0t/\hbar}+\text{h.c.}\\&=\frac{1}{\hbar^2}\sum_{i,j=1}^{2}\sum_{\nu,\mu=3}^{D}\sum_{\bar{\omega}_{i\nu}}\sum_{\bar{\omega}_{j\mu}}\Gamma^{T_1}(\bar{\omega}_{i\nu})\left[A_{i\nu}(\bar{\omega}_{i\nu})\rho(t)A^\dagger_{j\mu}(\bar{\omega}_{j\mu})-A^\dagger_{j\mu}(\bar{\omega}_{j\mu})A_{i\nu}(\bar{\omega}_{i\nu})\rho(t)\right]+\text{h.c.}\end{aligned} \tag{S12}$$

with the damping rate given by

$$\Gamma^{T_1}(\bar{\omega}_{i\nu})=\begin{cases}\pi\sum_{\mathbf{k},p}g^2_{\mathbf{k}p}n^{T_1}_{\mathbf{k}p}\delta(\omega_{i\nu}+\omega_{\mathbf{k}p})=\gamma\hbar^2n^{T_1}_{\omega_{i\nu}}, & \text{when}\quad\bar{\omega}_{i\nu}<0\\ \pi\sum_{\mathbf{k},p}g^2_{\mathbf{k}p}(n^{T_1}_{\mathbf{k}p}+1)\delta(\omega_{i\nu}-\omega_{\mathbf{k}p})=\gamma\hbar^2(n^{T_1}_{\omega_{i\nu}}+1), & \text{when}\quad\bar{\omega}_{i\nu}>0\end{cases} \tag{S13}$$

Under rotating wave approximation (RWA) the dissipation term can be written as

$$
\begin{aligned}
\mathscr{D}^{T_1}(\rho) &= \frac{1}{\hbar^2}\sum_{i,j=1}^{2}\sum_{\nu,\mu=3}^{D}\sum_{\bar{\omega}_{i\nu}\bar{\omega}_{j\mu}>0}\Gamma^{T_1}(\bar{\omega}_{i\nu})\left[A_{i\nu}(\bar{\omega}_{i\nu})\rho(t)A^{\dagger}_{j\mu}(\bar{\omega}_{j\mu})-A^{\dagger}_{j\mu}(\bar{\omega}_{j\mu})A_{i\nu}(\bar{\omega}_{i\nu})\rho(t)\right]+\text{h.c.} \\
&= \frac{1}{\hbar^2}\sum_{i,j=1}^{2}\sum_{\nu,\mu=3}^{D}\Gamma^{T_1}(\bar{\omega}_{i\nu}>0)(U^T_{\nu,i+2}+U^T_{\nu,i+4})(U_{j+2,\mu}+U_{j+4,\mu})\left(|i\rangle\langle\underline{\nu}|\rho|\underline{\mu}\rangle\langle j|-|\underline{\mu}\rangle\langle j|i\rangle\langle\underline{\nu}|\rho\right) \\
&\quad+\frac{1}{\hbar^2}\sum_{i,j=1}^{2}\sum_{\nu,\mu=3}^{D}\Gamma^{T_1}(\bar{\omega}_{i\nu}<0)(U_{i+2,\nu}+U_{i+4,\nu})(U^T_{\mu,j+2}+U^T_{\mu,j+4})\left(|\underline{\nu}\rangle\langle i|\rho|j\rangle\langle\underline{\mu}|-|j\rangle\langle\underline{\mu}|\underline{\nu}\rangle\langle i|\rho\right) \\
&\quad+\text{h.c.}+\frac{1}{\hbar^2}\sum_{i,j=1}^{2}\sum_{\nu,\mu=3}^{D}\sum_{p,q=3}^{D}\Gamma^{T_1}(\bar{\omega}_{i\nu}<0)(U_{i+2,\nu}+U_{i+4,\nu})(U^T_{\mu,j+2}+U^T_{\mu,j+4})U^T_{\nu p}U_{q\mu} \\
&\qquad\times(|p\rangle\langle i|\rho|j\rangle\langle q|-|j\rangle\langle q|p\rangle\langle i|\rho)+\text{h.c.} \\
&= \gamma\sum_{i,j=1}^{2}\sum_{\nu,p=3}^{D}\sum_{f=2,4}(U^T_{\nu,i+2}+U^T_{\nu,i+4})(U_{j+2,\mu}+U_{j+4,\mu})U_{p\nu}\left[\left(n^{T_1}_{\omega_{i\nu}}+1\right)\left(\sigma^-_{ip}\rho\sigma^+_{j+f,j}-\sigma^+_{j+f,j}\sigma^-_{ip}\rho\right)\right. \\
&\qquad\left.+n^{T_1}_{\omega_{i\nu}}\left(\sigma^+_{pi}\rho\sigma^-_{j,j+f}-\sigma^-_{j,j+f}\sigma^+_{pi}\rho\right)\right]+\text{h.c.}
\end{aligned}
\tag{S14}
$$

For the coupling of system to low-frequency fluctuations of proteins, the dipole moment of system takes the form of

$$
\sum_{n=3}^{D}\bar{\gamma}_n\sigma_{nn}=\sum_{i=3}^{D}\sum_{j=3}^{D}\sum_{k=3}^{D}\bar{\gamma}_iU_{ik}U^T_{ji}|\underline{k}\rangle\langle\underline{j}| \tag{S15}
$$

which gives the interaction between system and low-fluctuation environment in the interaction picture

$$
\begin{aligned}
\tilde{H}^{(2)}_{int}(t) &= \sum_{i=3}^{D}\sum_{j=3}^{D}\sum_{k=3}^{D}\bar{\gamma}_iU_{ik}U^T_{ji}|\underline{k}\rangle\langle\underline{j}|e^{-i\omega_{kj}t}\otimes C(t) \\
C(t) &= \sum_{\mathbf{q},s}f_{\mathbf{q}s}\left(b_{\mathbf{q}s}e^{-i\nu_{\mathbf{q}s}t}+b^{\dagger}_{-\mathbf{q},s}e^{i\nu_{\mathbf{q}s}t}\right)
\end{aligned}
\tag{S16}
$$

with the correlation function

$$
\langle C(t)C(t-s)\rangle=\sum_{\mathbf{q},\sigma}f^2_{\mathbf{q}\sigma}\left[\left(n^{T_2}_{\mathbf{q}\sigma}+1\right)e^{-i\nu_{\mathbf{q}\sigma}s}+n^{T_2}_{\mathbf{q}\sigma}e^{i\nu_{\mathbf{q}\sigma}s}\right] \tag{S17}
$$

Through the similar procedures as what has been carried out for radiations, we obtain the dissipation term contributed by the low-frequency fluctuations

$$
\begin{aligned}
\mathscr{D}^{T_2}(\rho) &= \frac{1}{\hbar^2}\text{Tr}_Be^{-iH_0t/\hbar}\int_0^t\mathrm{d}s\left[\tilde{H}^{(2)}_{int}(t-s)\tilde{\rho}(t)\rho_R(0)\tilde{H}^{(2)}_{int}(t)-\tilde{H}^{(2)}_{int}(t)\tilde{H}^{(2)}_{int}(t-s)\tilde{\rho}(t)\rho_R(0)\right]e^{iH_0t/\hbar}+\text{h.c.} \\
&= \sum_{a,b=3}^{D}\sum_{i,c=3}^{D}\sum_{\mu,\nu=3}^{D}\bar{\gamma}_i\bar{\gamma}_c\Gamma^{T_2}_{\nu\mu}U_{i\nu}U^T_{\mu i}U^T_{\nu a}U_{b\mu}\left(\sigma_{ab}\rho\sigma_{cc}-\sigma_{cc}\sigma_{ab}\rho\right)+\text{h.c.}
\end{aligned}
\tag{S18}
$$

where

$$
\begin{aligned}
\Gamma^{T_2}_{\nu\mu} &= \int_0^{\infty}\frac{\mathrm{d}\omega}{\pi}S(\omega)\left[n^{T_2}_{\omega}\delta(\omega+\omega_{\nu\mu})+\left(n^{T_2}_{\omega}+1\right)\delta(\omega-\omega_{\nu\mu})\right] \\
&= \begin{cases} S(-\omega_{\nu\mu})n^{T_2}_{-\omega_{\nu\mu}}, & (\nu>\mu) \\ \frac{2E_R}{\pi\hbar}\frac{k_BT_2}{\hbar\omega_d}, & (\nu=\mu) \\ S(\omega_{\nu\mu})\left(n^{T_2}_{\omega_{\nu\mu}}+1\right), & (\nu<\mu) \end{cases}
\end{aligned}
\tag{S19}
$$

With the dissipations contributed by the two environments, shown in Eq.(S14) and (S18), one can reach the operator master equation for the system

$$\frac{d\rho}{dt}=\frac{i}{\hbar}[\rho,H_S]+\gamma\sum_{i,j=1}^{2}\sum_{\nu,p=3}^{6}\sum_{f=2,4}\left(U^T_{\nu,i+2}+U^T_{\nu,i+4}\right)U_{p\nu}\left[\left(n^{T_1}_{\omega_{i\nu}}+1\right)\right.$$
$$\times\left(\sigma^-_{ip}\rho\sigma^+_{j+f,j}-\sigma^+_{j+f,j}\sigma^-_{ip}\rho\right)+n^{T_1}_{\omega_{i\nu}}\left.\left(\sigma^+_{pi}\rho\sigma^-_{j,j+f}-\sigma^-_{j,j+f}\sigma^+_{pi}\rho\right)\right] \tag{S20}$$
$$+\sum_{a,b=3}^{6}\sum_{i,c=3}^{6}\sum_{\mu,\nu=3}^{6}\bar{\gamma}_i\bar{\gamma}_c\Gamma^{T_2}(\omega_{\nu\mu})U_{i\nu}U^T_{\mu i}U^T_{\nu a}U_{b\mu}\times(\sigma_{ab}\rho\sigma_{cc}-\sigma_{cc}\sigma_{ab}\rho)+\text{h.c.}$$

where U is the orthogonal matrix diagonalizing the Hamilton matrix in Eq.(S5) and $\hbar\omega_{nm}\equiv E_m-E_n$. $n^T_\omega=[\exp(\hbar\omega/k_BT)-1]^{-1}$ is the Bose occupation of frequency ω at temperature T. For the protein environment, we use the Debye spectral density: $S(\omega)=(2E_R/\pi\hbar)(\omega\omega_d/(\omega^2+\omega_d^2))$, where E_R is so-called reorganization energy and $\Gamma^{T_2}_{\nu\mu}=S(|\omega_{\nu\mu}|)n^{T_2}_{|\omega_{\nu\mu}|}$ for $\nu>\mu$, $S(|\omega_{\nu\mu}|)(n^{T_2}_{|\omega_{\nu\mu}|}+1)$ for $\nu<\mu$, or $(2E_R/\pi\hbar)(k_BT_2/\hbar\omega_d)$ for $\nu=\mu$.

Control Models

To quantify the contribution of these vibronational coherences, it is essential for us to propagate a comparison between our model introduced above and the models in adiabatic regime (no exciton-vibron coupling), including the cases with and without the incoherent radiation environment. The models for control are denoted as *Model 1, Model 2* and *Model 3* where *Model 1* was included in main text.

1. *Model 1*: in adiabatic regime, including the incoherent radiations. Initially $\rho(0)=|0\rangle\langle 0|$. The contribution of ground-state vibronic coherence vanishes and the scaled probability on pigment B is defined as $\bar{P}^0_B(t)=P^0_B/(P^0_A+P^0_B)$ with $P^0_i=\langle i|\rho|i\rangle$; $i=A,B$. The only coherence is of the electronic type: $C^0_{ele}=\langle A|\rho|B\rangle$.
2. *Model 2*: in non-adiabatic regime but without including the incoherent radiations. Initially $\rho(0)=|A,0\rangle\langle A,0|$ according to the femtosecond experiments. The QME for this case is obtained by directly getting rid of the radiation terms (with the index T_1) in Eq.(8) in the main text and we ignore the details to avoid redundancy.
3. *Model 3*: in adiabatic regime and without including the incoherent radiations. Initially $\rho(0)=|A\rangle\langle A|$, according to the femtosecond experiments.

our model and *Model 1* lead to the nonequilibrium steady state with time-irreversibility while in *Model 2,3* the time-reversal symmetry is protected at steady state. Such difference will affect the role of excitonic coherence on excitation energy transfer, as will be elucidated as follows.

For *Model 1* it is straightforward to obtain the QME, by removing the exciton-vibron coupling

$$\frac{d\rho}{dt}=\frac{i}{\hbar}[\rho,H_S^{(1)}]+\gamma\sum_{\nu,\mu=2}^{3}\sum_{p,q=2}^{3}(U^T_{\nu 2}+U^T_{\nu 3})U_{p\nu}\left[(n^{T_1}_{\omega_{1\nu}}+1)\left(\sigma^-_{1p}\rho\sigma^+_{q1}-\rho\sigma^+_{q1}\sigma^-_{1p}\rho\right)\right.$$
$$+n^{T_1}_{\omega_{1\nu}}\left.\left(\sigma^+_{p1}\rho\sigma^-_{1q}-\sigma^-_{1q}\sigma^+_{p1}\rho\right)\right]+\sum_{a,b=2}^{3}\sum_{i,c=2}^{3}\sum_{\mu,\nu=2}^{3}\bar{\gamma}_i\bar{\gamma}_c\Gamma^{T_2}_{\nu\mu}U_{i\nu}U^T_{\mu i}U^T_{\nu a}U_{b\mu}\left(\sigma_{ab}\rho\sigma_{cc}-\sigma_{cc}\sigma_{ab}\rho\right)+\text{h.c.} \tag{S21}$$

where the Hamiltonian $H_S^{(1)}$ is of the 3×3 matrix

$$H_S^{(1)}=\begin{pmatrix}0&0&0\\0&\varepsilon_1&J\\0&J&\varepsilon_2\end{pmatrix} \tag{S22}$$

In *Model 2* where incoherent environment is removed, the QME for this case in non-adiabatic regime is obtained by directly getting rid of the radiation terms in Eq.(8) in the main text

$$\frac{d\rho}{dt}=\frac{i}{\hbar}[\rho,H_S^{(2)}]+\sum_{a,b=1}^{4}\sum_{i,c=1}^{4}\sum_{\mu,\nu=1}^{4}\bar{\gamma}_i\bar{\gamma}_c\Gamma^{T_2}_{\nu\mu}U_{i\nu}U^T_{\mu i}U^T_{\nu a}U_{b\mu}\left(\sigma_{ab}\rho\sigma_{cc}-\sigma_{cc}\sigma_{ab}\rho\right)+\text{h.c.} \tag{S23}$$

where $H_S^{(2)}$ in Eq.(S23) takes the lower block of the Hamilton matrix H_S in Eq.(S5), which is 4×4 matrix.

In *Model 3*, $H_S^{(3)}$ is lower block of $H_S^{(1)}$ in Eq.(S22) and the subsequent QME reads

$$\begin{aligned}
\dot\rho_{AA} &= \frac{i\Delta}{\hbar}(\rho_{AB}-\rho_{BA})\,; \quad \dot\rho_{BB} = -\frac{i\Delta}{\hbar}(\rho_{AB}-\rho_{BA}) \\
\dot\rho_{AB} &= \left[\frac{i\Delta}{\hbar}+(\bar\gamma_1-\bar\gamma_2)R_{21}\right]\rho_{AA} - \left[\frac{i\Delta}{\hbar}+(\bar\gamma_1-\bar\gamma_2)R_{12}\right]\rho_{BB} + \left[\frac{i}{\hbar}(\varepsilon_B-\varepsilon_A)+(\bar\gamma_1-\bar\gamma_2)(R_{22}-R_{11})\right]\rho_{AB} \\
\dot\rho_{BA} &= \left[\frac{\Delta}{i\hbar}+(\bar\gamma_1-\bar\gamma_2)R_{21}\right]\rho_{AA} - \left[\frac{\Delta}{i\hbar}+(\bar\gamma_1-\bar\gamma_2)R_{12}\right]\rho_{BB} + \left[\frac{\varepsilon_B-\varepsilon_A}{i\hbar}+(\bar\gamma_1-\bar\gamma_2)(R_{22}-R_{11})\right]\rho_{BA} \\
&\text{and} \\
R_{ab} &= \sum_{i,\mu,\nu=1}^{2}\bar\gamma_i\Gamma^{T_2}_{\nu\mu}U_{i\nu}U^T_{\mu i}U^T_{\nu a}U_{b\mu},\ \ a,b=1,2
\end{aligned} \tag{S24}$$

and the equations governing the time evolution of electronic coherence are obtained by eliminating the population terms

$$\begin{aligned}
\ddot\rho_{AB} &= (-P+iQ)(\rho_{AB}-\rho_{BA})-(R+iS)\dot\rho_{AB} \\
\ddot\rho_{BA} &= (P+iQ)(\rho_{AB}-\rho_{BA})-(R-iS)\dot\rho_{BA} \\
&\text{and} \\
P &= -\frac{2\Delta^2}{\hbar^2},\ Q=\frac{i\Delta}{\hbar}(\bar\gamma_1-\bar\gamma_2)(R_{12}+R_{21}),\ R=(\bar\gamma_1-\bar\gamma_2)(R_{11}-R_{22}),\ S=\frac{i(\varepsilon_A-\varepsilon_B)}{\hbar}
\end{aligned} \tag{S25}$$

and then $\ddot r = -R\dot r + S\dot s - 2Qs$, $\ddot s = -R\dot s - S\dot r - 2Ps$ where $\rho_{AB} = r+is$. The eigensolution is $r = u_0 e^{\Gamma t}$, $s = v_0 e^{\Gamma t}$, which leads to

$$\left\{\begin{aligned} &(\Gamma^2+\Gamma R)u_0-(\Gamma S-2Q)v_0=0 \\ &\Gamma S u_0+(\Gamma^2+\Gamma R+2P)v_0=0 \end{aligned}\right. \Longrightarrow \Gamma_0=0 \ \text{and}\ (\Gamma_n+R)(\Gamma_n^2+\Gamma_n R+2P)+S(\Gamma_n S-2Q)=0 \tag{S26}$$

Hence the solution is

$$\begin{pmatrix} r(t) \\ s(t)\end{pmatrix} = \begin{pmatrix} u_0 \\ 0\end{pmatrix} + \sum_{n=1}^{3}\begin{pmatrix} 1 \\ \frac{\Gamma_n^2+\Gamma_n R}{\Gamma_n S-2Q}\end{pmatrix} u_n e^{\Gamma_n t}, \quad \text{with} \begin{pmatrix} r(0) \\ s(0)\end{pmatrix}=0,\ \begin{pmatrix} \dot r(0) \\ \dot s(0)\end{pmatrix} = \begin{pmatrix} (\bar\gamma_1-\bar\gamma_2)R_{21} \\ \frac{\Delta}{\hbar}\end{pmatrix} \tag{S27}$$

with $\Gamma_2=\Gamma_1^*$, $\Gamma_3\in\mathbb{R}$ and $u_2=u_1^*$, $u_3\in\mathbb{R}$ due to the reality of r and s. The initial condition is given by $\rho(0)=|A\rangle\langle A|$ according to the femtosecond experiments and it gives rise to the following matrix equation

$$\begin{pmatrix} 1 & 1 & 1 & 1 \\ 0 & \frac{\Gamma_1^2+\Gamma_1 R}{\Gamma_1 S-2Q} & \frac{\Gamma_2^2+\Gamma_2 R}{\Gamma_2 S-2Q} & \frac{\Gamma_3^2+\Gamma_3 R}{\Gamma_3 S-2Q} \\ 0 & \Gamma_1 & \Gamma_2 & \Gamma_3 \\ 0 & \frac{\Gamma_1^3+\Gamma_1^2 R}{\Gamma_1 S-2Q} & \frac{\Gamma_2^3+\Gamma_2^2 R}{\Gamma_2 S-2Q} & \frac{\Gamma_3^3+\Gamma_3^2 R}{\Gamma_3 S-2Q}\end{pmatrix}\begin{pmatrix} u_0 \\ u_1 \\ u_2 \\ u_3\end{pmatrix} = \begin{pmatrix} 0 \\ 0 \\ (\bar\gamma_1-\bar\gamma_2)R_{21} \\ \frac{\Delta}{\hbar}\end{pmatrix} \tag{S28}$$

to determine u_n's.

Survival of coherence by including two vibrational modes

In the main text, we provide the figures for the coherence dynamics while including one vibrational mode. Here we will provide the figures (Fig.1) illustrating the effect of multi-vibrational modes. By taking into account two vibrational modes, the electronic coherence becomes much longer-lived. This shows that the surviving time of coherence by including two vibrational modes is around 10 times longer than that by including one mode.

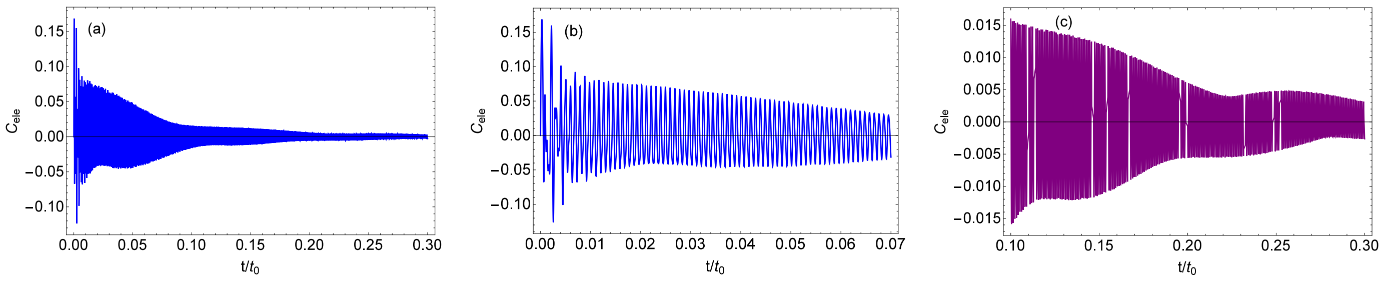

Figure 1. (Color online) Time evolution of electronic coherence for the cases including two vibrational modes. (b) and (c) are the zooming-in of coherence dynamics in different subintervals of time. The parameters are the same as in Fig.1 in main text.

Coherence dynamics for the cases without including incoherent environment

In the main text, we investigated the role of vibrational and ground-state coherences on the long-lived oscillation of excitonic coherence and quantified the effectiveness of the models previously used. Here we include the figures (Fig.2) for the coherence dynamics of the cases without considering the incoherent environment, in both non-adiabatic and adiabatic regimes. As we can see, the models only including the low-frequency fluctuations as bath are effective for describing the transient dynamics of coherences, in a qualitative manner.

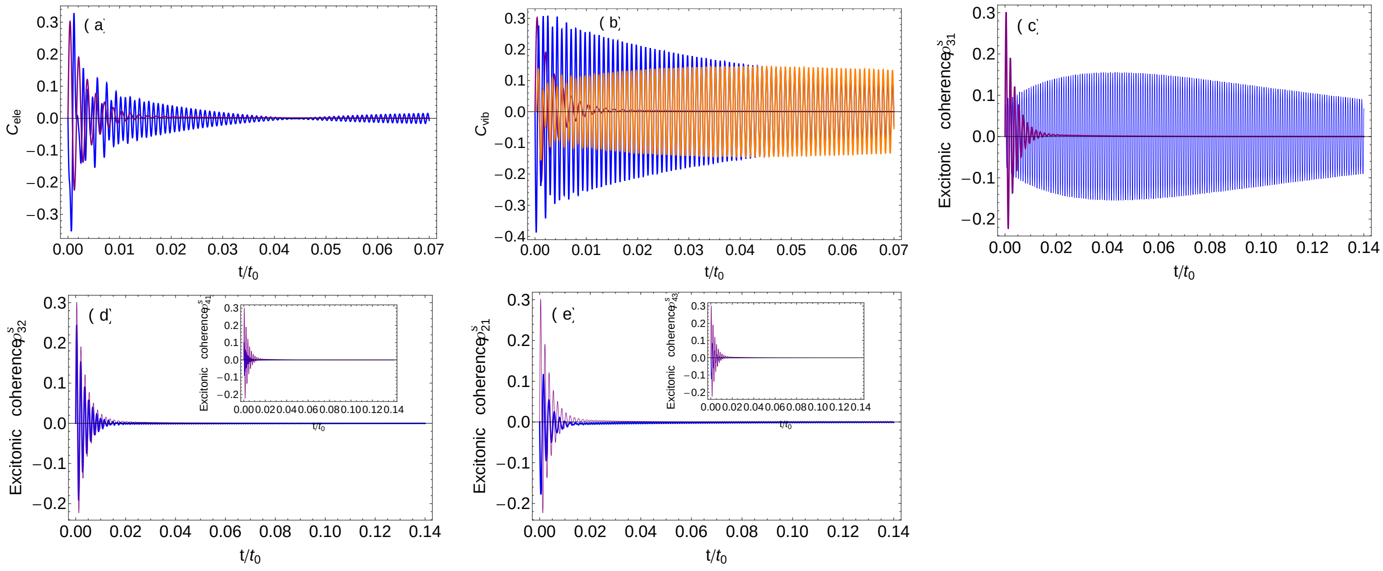

Figure 2. (Color online) Time evolution of coherence for the cases without including the incoherent environment. (a) and (b) are for the electronic and vibrational coherences in localized basis, respectively; (c,d,e) are the excitonic coherence in delocalized basis; In (a,b) the purple line corresponds to only electronic coherence in adiabatic regime while the blue line is for the non-adiabatic regime; In (b) blue and orange lines are for excited-state vibrational wave packets $\langle A,0|\rho|A,1\rangle$ and $\langle B,0|\rho|B,1\rangle$, respectively; In (c,d,e) the blue and purple lines are for the non-adiabatic and adiabatic regimes, respectively. The parameters are the same as in Fig.1 in main text.

In fact, our model proposed in Sec.II on the other hand, provides a test of the validity of *Model 2* popularly used in previous references where the incoherent radiations are not considered and only the low-frequency fluctuations of protein are included as bath. Particularly, the descriptions of the role of vibrational coherence on the long-lived electronic wave packet are in a similar way as that in our model above, by comparing Fig.2(a) with 2(a), 2(b) with 2(b) correspondingly. Moreover from the comparison of Fig.2(c), 2(d) and 2(e) in correspondence with Fig.2(d), 2(e) and 2(f), it is known that the dephasing of excitonic coherence (delocalized) and the pathways of coherent energy transfer are predicted in agreement with our model. *The role of ground-state coherence, however, cannot be captured by Model 2, which results in the failure of predicting the enhancement of excitation enery transfer reflected by population dynamics*.

The quantitative difference on the behaviors of coherence dynamics between *Model 2* and our model is because of (i) different initial conditions (in our case $\rho(0) = |0,0\rangle\langle 0,0|$ while in *Model 2* $\rho(0) = |A,0\rangle\langle A,0|$) and (ii) different regimes of steady state in long time limit. For (ii), our model will drive the system to the steady state with time-irreversibility while *Model 2* will alternatively drive the system to the steady state protecting the time-reversile. The time-irreversibility at steady state, as pointed out in Ref.[32] in the main text, does affect the dephasing behavior in a quantitative rather than qualitative way.
